# A general and efficient strategy for generating the stable enzymes

**DOI:** 10.1038/srep33797

**Published:** 2016-09-26

**Authors:** Xiao-Fei Zhang, Guang-Yu Yang, Yong Zhang, Yuan Xie, Stephen G. Withers, Yan Feng

**Affiliations:** 1 State Key Laboratory of Microbial Metabolism, School of Life Sciences and Biotechnology, Shanghai Jiao Tong University, Shanghai 200240, China; 2 Department of Chemistry, University of British Columbia Vancouver, British Columbia V6T 1Z1, Canada

## Abstract

The local flexibility of an enzyme’s active center plays pivotal roles in catalysis, however, little is known about how the flexibility of these flexible residues affects stability. In this study, we proposed an active center stabilization (ACS) strategy to improve the kinetic thermostability of *Candida rugosa* lipase1. Based on the B-factor ranking at the region ~10 Å within the catalytic Ser209, 18 residues were selected for site-saturation mutagenesis. Based on three-tier high-throughput screening and ordered recombination mutagenesis, the mutant VarB3 (F344I/F434Y/F133Y/F121Y) was shown to be the most stable, with a 40-fold longer in half-life at 60 °C and a 12.7 °C higher *T*_m_ value than that of the wild type, without a decrease in catalytic activity. Further analysis of enzymes with different structural complexities revealed that focusing mutations on the flexible residues within around 10 Å of the catalytic residue might increase the success rate for enzyme stabilization. In summary, this study identifies a panel of flexible residues within the active center that affect enzyme stability. This finding not only provides clues regarding the molecular evolution of enzyme stability but also indicates that ACS is a general and efficient strategy for exploring the functional robustness of enzymes for industrial applications.

Enzymes with highly effective and versatile catalytic functions are alternatives to conventional chemical catalysts^1^,^2^,^3^. The improvement of enzyme stability has attracted constant attention of researchers for a wide range of applications across a variety of fields. Extensive studies have revealed that the flexible segments of enzymes affect their stability due to increased fluctuation^4^,^5^,^6^,^7^,^8^. However, engineering the stability of enzymes remains a challenging work in terms of creating flexible segments with proper rigidity. The active center is a key component for catalytic functions^9^. Flexible residues within the active center of enzymes are essential for both catalysis and substrate binding, involving in the induced-fit interaction between enzymes and substrates^10^. However, little is known regarding their role in enzyme stability which provides a stable microenvironment for catalysis.

The B-factor (or B-value), indicating atomic displacement parameters obtained from X-ray data, is commonly used to represent residue flexibility^11^. Flexible residues, which have fewer contacts with other amino acids, may produce local perturbations inside highly complex networks of non-covalent connections. The residues trigger protein unfolding due to their large thermal fluctuations. Hence, introducing mutations to rigidify flexible residues may be an effective way to improve enzyme thermostability^12^. Based on the B-factor, Reetz *et al*.^13^ significantly increased the thermostability of lipase (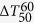 value of 45 °C) from *Bacillus subtilis* (LipA, 181 residues) (Fig. 1A), which is a minimal α/β hydrolase^14^. Later, Zhang *et al*.^15^ also reported a double-mutant of *Rhizomucor miehei* lipase (RML, 269 residues) based on B-factor design but only achieved a 1.24-fold thermostability increase. Damnjanović *et al*.^16^ mutated 12 residues with the highest B-factors on the surface of phosphatidylinositol-synthesizing *Streptomyces* phospholipase D (PLD, 509 residues), and the half-life of the best mutant was only 8.7 min longer compared to the parent (25.9 min). All of these results suggest that rigidifying flexible residues on larger enzymes based on only B-factor analysis, without considering other structural factors, may have a limitation in enhancing the stability of a variety of enzymes.

We questioned whether creating mutations in residues with the highest B-factors is efficient for small proteins but not suitable for large proteins, as almost all residues with the highest B-factors are located on the enzyme surface, which provide more solvent-accessible area than buried residues^17^. Considering that residues on the protein surface are far from the active center of large enzymes, simply reducing surface fluctuations may be insufficient for protecting the enzyme active center from heat-induced conformational changes^18^. In previous work, we attempted to enhance the kinetic stability of *Candida antarctica* lipase B (CalB, 317 residues) (Fig. 1B), by mutating the active center residues with the highest B-factors^19^,^20^. Indeed, these flexible residue cluster mutagenesis in the active center of CalB led to an efficient improvement on its stability. Crystal structures of CalB revealed that a new formed hydrogen bond network in the mutant D233G/L278M may rigidify a local flexible region in the vicinity of its active center^20^.

Due to the complexity of enzyme structures and the key role of the active center where substrate recognition and catalysis takes place, we propose a general active center stabilization (ACS) strategy to improve enzymes thermostability (Fig. 2). In this strategy, we pay more attentions to the flexible fragments rather than the flexible residues. Therefore, we divided the flexible fragments into two parts, outer phase and active center. There are no obvious boundaries in small proteins, whereas the obvious boundaries in large enzymes. Here, we have used a relatively larger lipase, *Candida rugosa* lipase1 (LIP1) containing 534 residues (Fig. 1C), as a model system to investigate the effect of ACS strategy. Besides of a canonical core with the α/β hydrolase fold domain, LIP1 possesses 10 helixes and 5 strands around the core structure as well as a lid segment covering its catalytic triad. LIP1 is supposed to present in an equilibrium between its open and closed states in solution^21^,^22^. The optimal temperature of LIP1 is 45 °C, and it loses nearly all activity after 60 °C incubation for 10 min^23^,^24^. Those residues with high B-factor ranking located within 10 Å of the catalytic residue Ser209 were chosen as candidates for site-saturated mutagenesis and ordered recombination mutagenesis (ORM). To avoid enzymes inactivation caused by active center mutagenesis, a smart three-tier screening procedure was set up to obtain the thermostability improved variants with relatively high activity. The kinetic and thermodynamic stability of the mutants were systematically characterized. In this study, the ACS strategy as an efficient methodology have been used to enhance the thermostability of a large lipase and it showed that mutagenesis focusing on flexible residues around 10 Å of the catalytic residue might significantly increase the success rate.

## Results

### Selection of the flexible residues in the active center for mutagenesis

The initial step of the ACS strategy was to select flexible residues. Indeed, critical catalytic residues were excluded to avoid any impairing on catalysis. To efficiently obtain stability improved LIP1 variants, those residues within 10 Å of catalytic residue Ser209 were considered as candidates for mutagenesis. The B-factors were analyzed using B-FITTER^25^ in two different structures of LIP1 (PDB codes: 1CRL and 1TRH), corresponding to the lipase ‘open’ and ‘closed’ states, respectively. Due to LIP1 structural complexity, the residues within 10 Å of catalytic residue Ser209 exhibit varied B-factor rankings as shown in the lower section of Fig. 3. Based on the B-factors analysis, eleven residues from each structure with the top highest B-factor ranking were selected, respectively, which resulted in total 22 candidate residues. However, four residues (Gly122, Gly123, Phe133, Leu302) are repeated in those candidate mutagenesis residues. Finally, only 18 residues of the top highest B-factor ranking were chosen around the active center for saturation mutagenesis. These residues were located in the α_12_ helix (Phe344, Phe345), the β_13_ strand (Phe434), the loop connecting the α_1_ helix and the β_5_ strand (Ser84, Lys85, Phe87, Glu88), the loop connecting the β_6_ strand and the α_3_ helix (Phe121, Gly122, Gly123, Gly124, Phe125, Glu126, Val127, Phe133), the loop connecting the α_10_ helix and the α_11_ helix (Phe296, Leu302), as well as a bend connecting the α_15_ helix and the α_16_ helix (Gly414). For efficient mutagenesis, the sites in cluster were combined for constructing one single library. Twelve libraries were constructed as following, library A (Phe344, Phe345), library B (Leu302), library C (Phe133), library D (Gly124, Phe125), library E (Glu126, Val127), library F (Phe121), library G (Phe87, Glu88), library H (Gly122, Gly123), library I (Ser84, Lys85), library J (Phe296), library K (Phe434), and library L (Gly414) (Fig. 3).

### Screening the mutants for both high thermostability and high catalytic activity

The recombinant plasmid pGAPZαA-*lip1* acted as the template to generate the saturation mutagenesis libraries using NNK degenerate primers (Supplementary Table S1). The LIP1 can be expressed in *P. pastoris* GS115 and secreted into the YPD culture medium. The criteria for selecting a mutation depended on its contribution to both thermostability and activity. The flowchart of the three-tier screening assay is depicted in Supplementary Fig. S1. The procedure includes a coarse screening on Petri dishes, which eliminates most of the inactive and less stable mutants, followed by a secondary screening in 96-well plates under highly stringent conditions^26^,^27^,^28^. Positives were further confirmed in four wells of the second 96-well plate assays and were sequenced^25^.

Finally, approximately 2.0 × 10^4^ clones in all mutant libraries were screened, accounting for about 95.6% library coverage of the variant space^29^,^30^,^31^. The thermostability of the mutant was estimated on fluorescent soft agar plates after exposure to 60 °C using the three-tier screening protocol. After screening, five mutants that showed improved thermostability were selected and sequenced to identify their mutations: F344M, F344I, F434Y, F133Y and F121Y (Supplementary Table S2, Fig. 4A). For all mutants, phenylalanine was substituted with other amino acids. Except for position 344, the other three positions have a tyrosine substitution (Fig. 4A). The 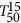, being the temperature at which 50% of the activity is lost after 15 min of incubation, for each of these mutants varied from 57.5 °C (F121Y, WT + 3.0 °C) to 62.4 °C (F344M, WT + 7.9 °C) (Fig. 4B). The half-lives of the mutants at 60 °C were enhanced 1.3- (F121Y, 7.8 min) to 5.1-fold (F344M, 30.9 min) compared to WT (6.0 min) (Fig. 4C). The catalytic efficiencies of the mutants were also higher than that of WT, except for F344M, which was similar to WT (Table 1).

To efficiently accumulate optimal mutations, we performed ORM, which combined all of the beneficial point mutants ordered from the best single mutation to the next site that showed lower improvement (Supplementary Table S2, Fig. 4A). Because the mutants F344M and F344I were mutated at the same position and have higher stability improvement than other beneficial point mutations, two paths for ORM were initiated for the F344M and F344I mutants. These two mutations were then combined with the F434Y mutant and renamed as VarA1 and VarB1, respectively, which exhibited significant improvements (VarA1, 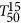 = 65.6 °C; VarB1, 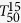 = 64.3 °C) (Fig. 4). For the next round of combination with F133Y, the resulting mutants VarA2 and VarB2 led to further improvements (VarA2, 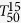 = 66.2 °C; VarB2, 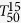 = 66.3 °C). Finally, the combination with F121Y resulted in two additional stable mutants, VarA3 (F344M/F434Y/F133Y/F121Y) (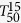 = 67.1 °C) and VarB3 (F344I/F434Y/F133Y/ F121Y) (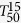 = 67.5 °C), representing remarkable increases in 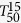 of 12.6 and 13 °C over WT, respectively. The optimal temperatures of the VarA3 and VarB3 mutants were increased from 45 °C to 60 °C and their half-lives were improved by 33-and 40-foldscompared to WT, respectively (Fig. 4C).

To confirm the thermal effects on the conformational stability of these mutants, we measured the *T*_m_ values of WT and the mutant enzymes by DSC assay (Fig. 4D). The results showed that the *T*_m_ values of the mutants were 4.5 °C (F434Y, 57.9 °C) to 17.4 °C (VarA2, 70.8 °C) higher compared to WT (53.4 °C). Notably, the *T*_m_ value of the mutant VarB3 with the highest 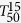 was lower than the mutant VarA2, indicating that thermodynamic stability are not necessarily consistent with kinetic stability of enzymes. Indeed, the lipase thermostability has been efficiently improved by the synergistic effect of the ORM.

### Catalytic kinetics and substrate specificity of WT and the mutants

To evaluate the catalytic functions of the mutants, their kinetic parameters were assessed using substrate *p*NP-C8 at 40 °C. As shown in Table 1, all single-site mutants retained their catalytic ability comparable to the wild-type (*k*_cat_/*K*_m_ from to 100 to 139%). The further combined mutants exhibited increases in *k*_cat_/*K*_m_ values, except for the VarB3 mutant (decreased 29.3%). Interestingly, the VarB1 mutant exhibited 2.2-fold higher activity compared to WT, whereas the most stable mutant, VarB3, exhibited a 26% increase in both *k*_cat_ and *K*_m_, resulting in an almost identical catalytic efficiency *k*_cat_/*K*_m_ compared to WT. These results indicated that the flexible residues in the active center exerted a great impact on both thermostability and catalytic activity.

The substrate spectra for various acyl chain lengths were also measured for all of the mutants at 40 °C, and the optimum substrate (*p*NP-C8) was the same as the parent (Fig. 5), suggesting that mutations in the flexible residues do not lead to changes in the catalytic spectra. It is possible that the replacement of the phenylalanine by neutral or aromatic residues did not change the conformation of the binding pocket and thus retained the substrate preference.

### Rigidity analysis of the best mutant, VarB3

To investigate the rigidity of the active center, the PredyFlexy web server was used to predict the normalized B-factor of the best mutant, VarB3^32^. The normalized B-factors of all of the selected flexible residues are listed in Supplementary Table S3. As shown in Fig. 6A, the VarB3 mutant led to an increase in the rigidity of the region from 122 to 136, the region from 343 to 345, and the region from 432 to 436. All of these regions are near the mutated sites of residues 121, 133, 344 and 434.

The modeling structure of the VarB3 mutant was constructed using Discover Studio3.5 and was assessed using PROCHECK analysis^33^, which revealed that only one residue (Ile18) was in a disallowed region in the Ramachandran Plot, similar to the wild type (Supplementary Fig. S3). We observed no gross changes in the structure of the VarB3 mutant compared to WT (Fig. 6B). The mutated positions at 121 and 133 were located in the same loop region from 120 to 136, and positions 344 and 434 were located in α12 and β13, respectively. However, the model structure showed that the Tyr133 residue formed 3 hydrogen bonds with two water molecules (W_a_ and W_b_) and Gly122, generating a new hydrogen-bond network (Fig. 7A). The Tyr121 residue formed 4 hydrogen bonds with water molecule W_c_, Phe128, Asn155 and Tyr156 (Fig. 7A). The new hydrogen-bond networks may enhance the stability of the loop region from 120 to 136. The Ile344 residue shortened the distance between Thr343 and Phe345, which resulted in the formation of a 3.3 Å hydrogen bond between them (Fig. 7B). Furthermore, the lengths of the 3 hydrogen bonds between Phe345 and Ser348, Gly346 and Ser349 (2 hydrogen bonds), respectively, became shorter from 3.3 Å to 3.2 Å. Compared to the structure of WT, the structural flexibility around position 344 was significantly increased, which was annotated by a prolonged α_12_ helix. The Tyr434 residue led to that the generation of a new hydrogen bond with the water molecule W_d_, and the distances of some hydrogen bonds around β_13_ were shortened, thus contributing to a stabilized LIP1 (Fig. 7C).

## Discussion

The evolutionary strategy for enzyme stabilization without compromising activity has important industrial value. In this study, we have used a large *C. rugosa* LIP1 as model enzyme to verify ACS strategy for thermostability improvement. Based on three-tier high-throughput screening and ORM, the most stable mutant, VarB3 (F344I/F434Y/F133Y/F121Y), exhibited a significant 40-fold increase in its half-life at 60 °C and a 12.7 °C higher *T*_m_ value, indicating that the ACS strategy applications could extend in extreme conditions.

Notably, all mutated residues are the aromatic acid phenylalanine in VarB3. Three (F344, F133 and F121) of these residues came from the ‘open’ structure and two (F434 and F133) were derived from the ‘closed’ structure. The B-factor ranking of F133 in the ‘open’ structure is higher than that in the ‘closed’ structure. Indeed, the unstable ‘open’ structures of lipases in polar (aqueous) environments have been identified by molecular dynamic analysis^34^. Hence, the more stabilized effect in ‘open’ lipases might come from a stability improvement on this structure which is crucial for catalysis and the kinetic stability of lipase. Furthermore, except for F344, the other positions were replaced by a polar tyrosine, which may work as a hydrogen bond donor for the nearby amino acids or water molecules^35^,^36^. It is known that intramolecular hydrogen bonding interactions play important roles in the proteins thermostability^37^,^38^.

To understand the structural alterations responsible for the improved kinetic thermostability, a modeling structure of VarB3 was constructed. In the structural model of VarB3, it shows that the both of mutated positions F133 and F121 locate within the loop region from 120 to 136, whereas the mutated F344 and F434 locate at the α_12_-helices and the β_13_-strand, respectively. In addition, the distance between F344 and F434 is large and no any direct interaction between them could be found. The stability improvement of the enzyme might benefit from the F344 and F434 single-point accumulated effects^39^. ORM was performed to rapidly determine the optimal combination of these beneficial point mutations, similar to iterative sited-directed mutagenesis (ISM)^25^. The combined results showed that the mutant VarA2 (F344I/F434Y) exhibited the greatest (2.2-fold) catalytic efficiency increase and a higher stability than other beneficial mutations. However, it is showed that the stability could be further increased by mutations combination, whereas catalytic efficiency was reduced in this process. The best thermostability improvement could be observed in the mutant VarB3, which also maintain a relatively high catalytic efficiency comparable to WT. This could be explained by the fact that the enzyme requires a reasonable trade-off between stability and activity in the active center^40^,^41^. Protein stability cannot be limitlessly increased, and proper conformation flexibility is critical for protein activity, whereas superposition tends to confer reduced flexibility of the residues at the substrate binding site, which results in a lower catalytic constant of the VarB3 mutant^42^.

Although the modeling structure of VarB3 was similar to that of the WT (Fig. 6B), the intramolecular interactions around the mutated sites exhibited subtle variations. Moreover, the prediction of the normalized B-factor also showed that the residues around the mutated positions had lower B-values compared with WT (Supplementary Table S3). Tyrosine substitutions at positons 121 and 133 formed two hydrogen-bond networks that strengthened the rigidity of the loop (Fig. 7A). Other mutated positions 344 and 434 resulted in new hydrogen bonds and closer packing around them (Fig. 7B,C). These intramolecular interactions play important roles in LIP1 stability by rigidifying the active center^43^.

**Figure 1:**
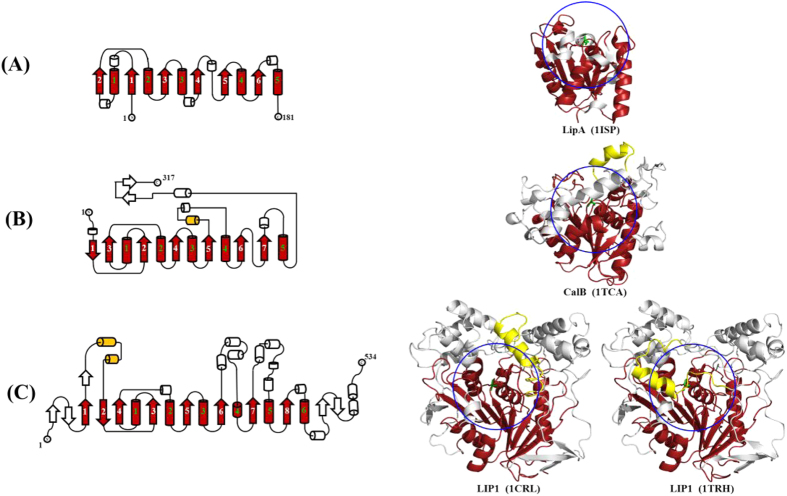
The topological structures of monomeric globular lipases LipA (**A**), CalB (**B**), and LIP1 (**C**). α-helices and β-strands are shown as cylinders and arrows, respectively. The canonical α/β-hydrolase folds are in red. The lids of LIP1 and CalB are shown in yellow. Other structures in the catalytic domains are shown in white. All serine catalytic residues are represented as green sticks. The blue rings represent the range of 10 Å around the catalytic residue serine.

**Figure 3:**
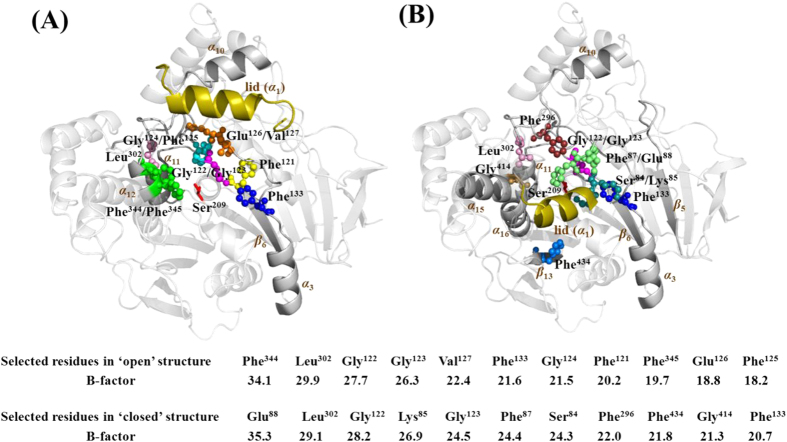
The sites in the active center of LIP1 that were chosen for saturation mutagenesis. The lower section shows the B-factors of all targeted sites. The structural models based on the open state (1CRL) (**A**) and closed state (1TRH) (**B**) of the LIP1 X-ray structures. Library (**A**) (green spheres), library (**B**) (pink spheres), library C (blue spheres), library D (cyan spheres), library E (orange spheres), library F (yellow spheres), library G (lime spheres), library H (magenta spheres), library I (deep teal spheres), library J (ruby spheres), library K (marine spheres), and library L (sand spheres). Catalytic residue Ser209 is shown in red, and the lid is colored in olive.

**Figure 4:**
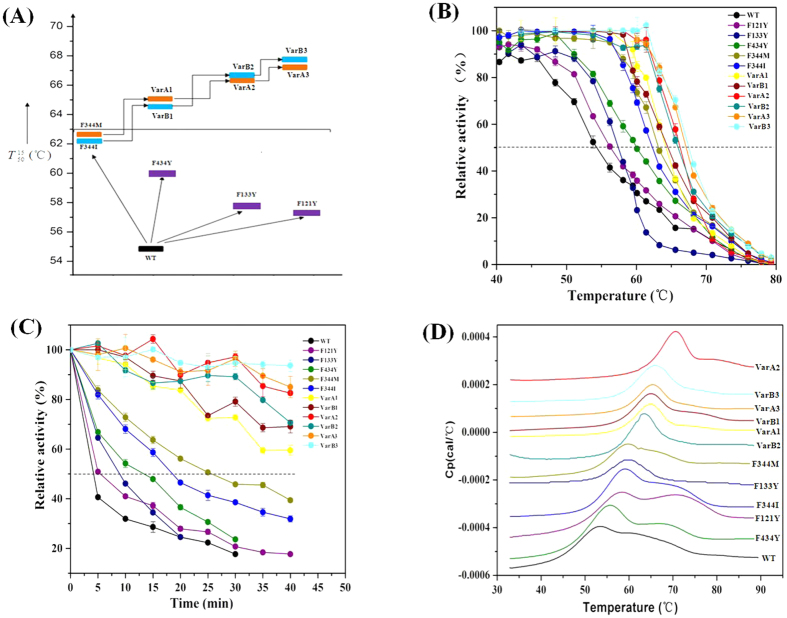
Thermostability of WT and all mutants. (**A**) Thermostability, expressed as 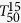 values (defined as the temperature at which the enzyme retains 50% of its activity after 15 min of incubation) of all mutants considered in this study: WT (black bar), F434Y, F133Y and F121Y (purple bar), F344M and F344M combination mutants (orange bar), and F344I and F444I combination mutants (blue bar). (**B**) Residual activity curves of WT and all mutants. (**C**) Thermostability at 60 °C. The activities of the enzymes without heat treatment were defined as 100%. (**D**) Thermal unfolding of WT and its mutants. The scans were performed at 60 °C/h over a temperature range of 30–90 °C using VP-cap DSC. The combinational mutants are VarA1 (F344M/F434Y), VarA2 (F344M/F434Y/F133Y), VarA3 (F344M/F434Y/F133Y/F121Y), VarB1 (F344I/F434Y), VarB2 (F344I/F434Y/F133Y), and VarB3 (F344I/F434Y/F133Y/F121Y).

**Figure 5:**
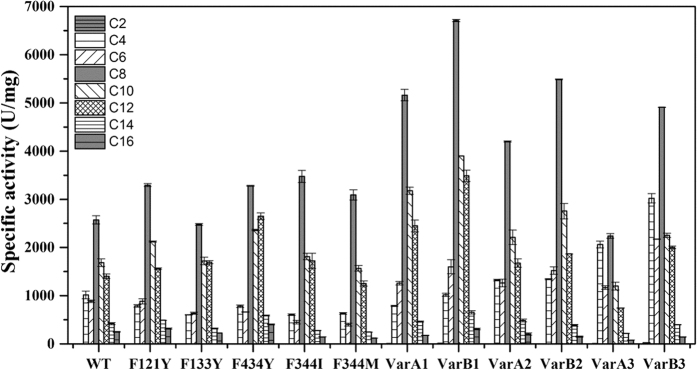
Substrate spectra analysis of WT and its mutants towards a variety of acyl chain-length *p*NP-esters. The hydrolysis activities of enzymes toward *p*NP-ester were determined at 40 °C with a 50 mM Tris-HCl, pH 8.0 buffer containing 0.1% (*w/v*) gum arabic and 0.2% (*w/v*) sodium deoxycholate.

To propose a general and efficient rule as guidance for enzyme stabilization, three model lipases were performed analysis, namely *Bacillus subtilis* LipA*, Candida antarctica* CalB and *Candida rugosa* LIP1 (in ‘open’ and ‘closed’ states). These enzymes share the same canonical α/β fold core structure, but exhibit different structural complexities (Fig. 1). As a minimal α/β hydrolase, LipA is the first enzyme to improve the thermostability based on B-factor^13^ and contains only 181 amino acids. Due to that most of the residues in LipA are located within 10 Å of the catalytic residue Ser77 (Fig. 1A), its thermostability improvement engineering can be regarded as a special case in the application of ACS strategy. For simplicity and clarity, only the effects of all single mutations on enzyme thermostability were evaluated. The other selected sites were found to exert no change in stability (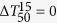). As shown in Fig. 8, the correlations among the distances of the mutated residues to the catalytic serine, the relative B-factor (percentage with the highest B-factor within 10 Å of the catalytic residue) and the effect on the enzyme thermostability (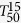) were analyzed. The mutated flexible residues with a 60–100 relative B-factor and within 10 Å of the catalytic serine were most effective in improving kinetic stability (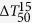) of enzymes. The mutations beyond ~14 Å and within ~6 Å did not improve stability.

To obtain a reliable conclusion for the active center residues correlated with enzymes thermostability, we have analyzed a number of published data of enzymes thermostability. On the basis of some of the most accurate data obtained to date, we have also identified certain positive correlations between the distance of the mutated residues to the catalytic residue and enzymes stability (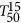, *t*_1/2_ or *T*_m_). As shown in the Supplementary Fig. S3, most of the enzymes with remarkably increased thermostability (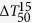 >3 °C, *t*_1*/2*_ > 85 folds and Δ*T*_*m*_  >3 °C) have similar trend of critical mutations located around 10 Å to catalytic residue. Hence, the analysis showed that the mutagenesis instead of targeting the entire enzyme, focusing mutagenesis on the flexible residues within 10 Å of the catalytic residue might significantly enhance their stability.

In summary, our study showed that ACS is a general and efficient strategy for enhancing thermostability. Analysis of the mutation results for enzymes with different structural complexities indicated that the highly flexible residues around 10 Å of the catalytic residue are more helpful for enzyme thermostability increases, which provide a guidance for improving the stability of a variety of industrial enzymes.

## Material and Methods

### Strains, plasmids, and materials

The *lip1* gene from *C. rugosa* ATCC14830 (JCM9586) was purchased from Japan Collection of Microorganisms (JCM). *E. coli* DH5α was used for the propagation and construction of a mutagenesis library. *P. pastoris* GS115 and the plasmid pGAPZαA (Invitrogen, Beijing, China) were the host and constitutive expression vector, respectively. The recombinant plasmid pGAPZαA-*lip1* used for saturation mutagenesis was constructed in our lab. PrimerSTAR Max DNA polymerase, restriction endonucleases, and polymerase chain reaction (PCR) reagents were purchased from TaKaRa (Dalian, China). A protein assay kit was purchased from BIOTEKE (Beijing, China). The antibiotic zeocin and Ni-nitrilotriacetate (NTA) were purchased from Invitrogen (Beijing, China) and GE Health (Uppsala, Sweden), respectively. All other chemicals and reagents were of analytical grade from Sigma (Sigma-Aldrich, USA).

### Methods to select the targets

We used the two different crystal structures of *C. rugosa* lipase1 in ‘open’ and ‘closed’ forms (PDB codes: 1CRL and 1TRH) from Protein Data Bank (http://www.rscb.org) to design a thermostable LIP1. The Pymol program was used to select the region within 10 Å of the catalytic residue Ser209. The average B-factors of residues in the selected region are calculated using the B-BITTER software^25^.

### LIP1 library generation

Site-saturation mutagenesis was performed using a pair of oligonucleotide primers. The target amino acid position was coded by NNK (sense strand) and MNN (antisense strand), where N = A, G, C or T, K = G or T and M = A or C. The whole-plasmid PCR method^44^ was performed using PrimerSTAR Max DNA polymerase with the pGAPZαA-*lip1* plasmid as the template DNA. The oligonucleotides used for site-saturation mutagenesis are listed in Supplementary Table S1. The PCR products were digested with *Dpn*I to remove the parent plasmid and purified using a PCR purification kit (Axygen). The resulting DNA mixture was transformed into *E. coli* DH5α competent cells. The cell suspension was spread on low Luria-Bertani (LLB) agar (1% tryptone, 0.5% yeast extract, 0.5% NaCl and 1.5% agar) containing zeoncin (25 μg/ml) plates and incubated at 37 °C for 1 day. All colonies (~10^4^/μg) were scraped, and the plasmids were extracted using a plasmid DNA preparation kit (Axygen). The mixed plasmids were linearized with *Avr*II restriction enzyme, and then transformed into *P. pastoris* GS115 competent cells via electroporation. The transformants were grown on YPD plates containing zeocin at 25 μg/ml at 30 °C for 3 days in order to select mainly single-copy transformants^45^.

### Three-tier screening of thermal stable mutants

The thermostability of mutants was assessed by following a three-tier screening protocol. Initially, coarse screening was performed on plates followed by a stringent screening in 96-well plate format. Three wells per plate were inoculated with WT as a control. Positives were further confirmed in four wells of another 96-well plate assay and were sequenced.

The transformants from the mutant library were replicated onto two similar YPD plates and the originals were stored. The duplicated plates were incubated for 24 h at 30 °C. Two sets of plates were brought back to room temperature followed by incubation of one set at 60 °C for 40 min, then cooling back to room temperature. A thin layer of fluorescent soft agar^45^ (YPD medium plate containing 5 g/l olive oil incorporated via ultrasonic emulsification and 10 mg/l rhodamine-B) was poured over the two sets of plates, followed by incubation at 37 °C for 5 h. The appearance of clear fluorescent halos around the colonies upon incubation indicated enzyme activity. Mutants showing clear fluorescent halos upon exposure to high temperatures were further screened for thermostability in 96-well plate format.

All of the mutants that showed high thermostability in the plate assay were inoculated in individual wells of 96-deep-well plates containing 800 μl of YPD medium including 25 μg/ml zeocin. After 72 h of growth at 30 °C, the cells were harvested by spinning the plates at 4,000 rpm for 20 min at 4 °C in a HITACHI centrifuge R5S4. The cultured supernatant in 96-deep-well plates was diluted fourfold using 50 mM Tris–HCl buffer (pH 7.5) to adjust the lipase activity. An aliquot of 10 μl of the diluted supernatant was added to 190 μl of substrate solution (0.2 mM *p*NP-C4, 50 mM Tris–HCl pH 8.0) at room temperature and agitated for 5 min. An aliquot of 100 μl of the diluted supernatant was added to 96-well PCR plates and heated to 58 °C for 15 min in a Mastercycler gradient PCR thermocycler (Eppendorf, Germany). After 10 min at 4 °C, cell debris and precipitated proteins were removed by centrifugation (4000 × g, 10 min), and the supernatant was used to determine residual activity.

To eliminate false positives, variants exhibiting more than 120% activity compared to heat-treated WT were transferred to four wells of new YPD-medium 96-deep-well plate for 72 h of culture. After a repeat of the same heat treatment, cells showing similar levels of thermostability in four wells were selected for sequencing.

### Protein expression and purification

A single colony of recombinant *P. pastoris* GS115 carrying the expression plasmid was initially inoculated into 4 ml of YPD medium containing 25 μg/ml zeocin and was cultured at 30 °C while shaking at 220 rpm until the optical density at 600  reached 1.0. The culture was then inoculated into 200 ml of YPD medium and was incubated at 30 °C for 72 h. Subsequently, the culture supernatant was harvested via centrifugation at 4,000 × g for 20 min, and then concentrated via ultrafiltration using a 10 kDa cut-off membrane (Millipore, USA). The obtained sample was dialyzed against 50 mM Tris-HCl at pH 7.5 and 500 mM NaCl buffer and loaded onto a Ni-nitriacetate (NTA) column. The proteins were eluted with a 200 mM imidazole buffer and dialyzed against 50 mM Tris-HCl buffer at pH 7.5. The protein samples were separated by 12% sodium dodecyl sulfate polyacrylamide gel electrophoresis (SDS-PAGE), and protein concentration was determined using a BCA protein assay (BIOTEKE, China) with bovine serum albumin (BSA) as the standard protein^46^.

### Enzyme activity assay

Lipolytic activity was measured using *p*-nitrophenyl ester as the substrate because hydrolysis liberates *p*-nitrophenol, which can be monitored by UV/Vis spectroscopy^47^. The standard assay was performed in a total volume of 1 ml containing 20 μl of 10 mM *p*NP-C8 in acetonitrile and 970 l of 50 mM, pH 8.0 Tris-HCl buffer containing 0.1% (*w/v*) gum arabic, 0.2% (*w/v*) sodium deoxycholate and 10 μl of enzyme solution^48^. After incubation at 40 °C for 5 min, the reaction was initiated by adding the corresponding enzymes, and the released *p*NP (ε = 16,000 M^−1^ cm^−1^) was measured at 405 nm using a UV-2550 spectrophotometer (Shimadzu, Japan). One unit of enzyme activity is defined as 1 μmol *p*NP liberated per minute.

### Analysis of biochemical properties

The 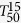 value was used to assess the enzyme thermal stability, defined as the temperature at which the enzyme retains 50% of its activity after 15 min of incubation. The purified enzyme solution (50 μl, 100 μg/ml) was incubated at a temperature gradient between 40 °C and 80 °C using a PCR thermo cycler for 15 min and cooled at 4 °C for 10 min followed by equilibration at 25 °C for 20 min. Samples were centrifuged to remove any aggregated protein before assaying for enzyme activity under standard assay conditions. The precise value was obtained by determining the inflection point of a fit of the residual activities at certain temperatures to a sigmoidal plot (sigmoidal Boltzmann fit using Origin 8.0). The half-life of enzyme inactivation (*t*_1/2_) was determined through incubation in Tris-HCl buffer (pH 7.5, 50 mM) for designated periods of time at 60 °C using the following equation^49^: *t*_1⁄2_ = ln 2/*k*_*d*_. *k*_d_ is the first-order rate constants determined by linear regression of ln(residual activity) *versus* the incubation time (*t*). The effect of temperature on the activity of LIP1 and its mutants was determined from 30 to 75 °C.

The kinetic parameters (*K*_m_, *k*_cat_ and *k*_cat_/*K*_m_) of the wild-type LIP1 and mutants were determined by increasing the *p*NP-C8 substrate concentration from 4 μM to 200 μM. To determine the substrate specificity, the hydrolysis rates of *p*NP-acetate (C2), *p*NP-butyrate (C4), *p*NP-hexanoate (C6), *p*NP-octanoate (C8), *p*NP-decanoate (C10), *p*NP-dodecanoate (C12), *p*NP-myristate (C14), and *p*NP-palmitate (C16) were monitored at 40 °C according to the standard assay.

### DSC analysis

Differential scanning calorimetry (DSC) was performed using a VP-cap DS caloimeter (MicroCal, Inc. Northampton, MA) at a scanning rate of 1 °C/min over a temperature range from 30 °C to 90 °C. The concentrations of proteins were controlled at 0.5 mg/ml dissolved in 10 mM PBS (pH 7.5). Before each DSC heating scan, the sample solutions were maintained at a starting temperature for 15 min. The melting temperature, *T*_m_, obtained from the DSC experiments corresponded to the maximum of the transition peak.

### Prediction of normalized B-factors and structural modeling of mutants

**Figure 6:**
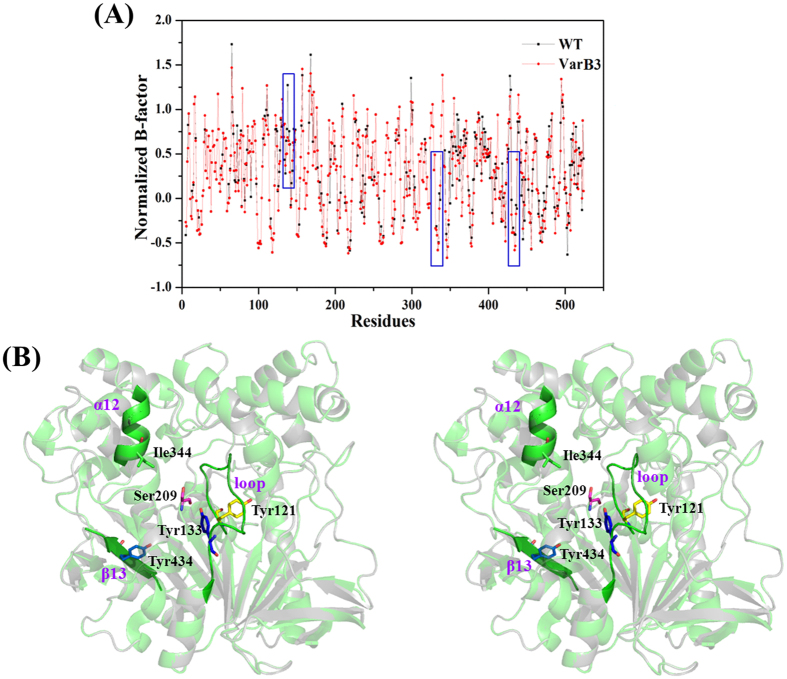
(**A**) Prediction of normalized B-factors of WT (black) and the VarB3 mutant (red) and (**B**) Stereoview of the superimposed structures of WT (gray) and the VarB3 mutant (green). Residues Y121, Y133, I344 and Y434 are labeled in yellow, blue, green and marine, respectively. The catalytic residue Ser209 is labeled in pink. Symbols: red, oxygen atom; blue, nitrogen atom.

**Figure 7:**
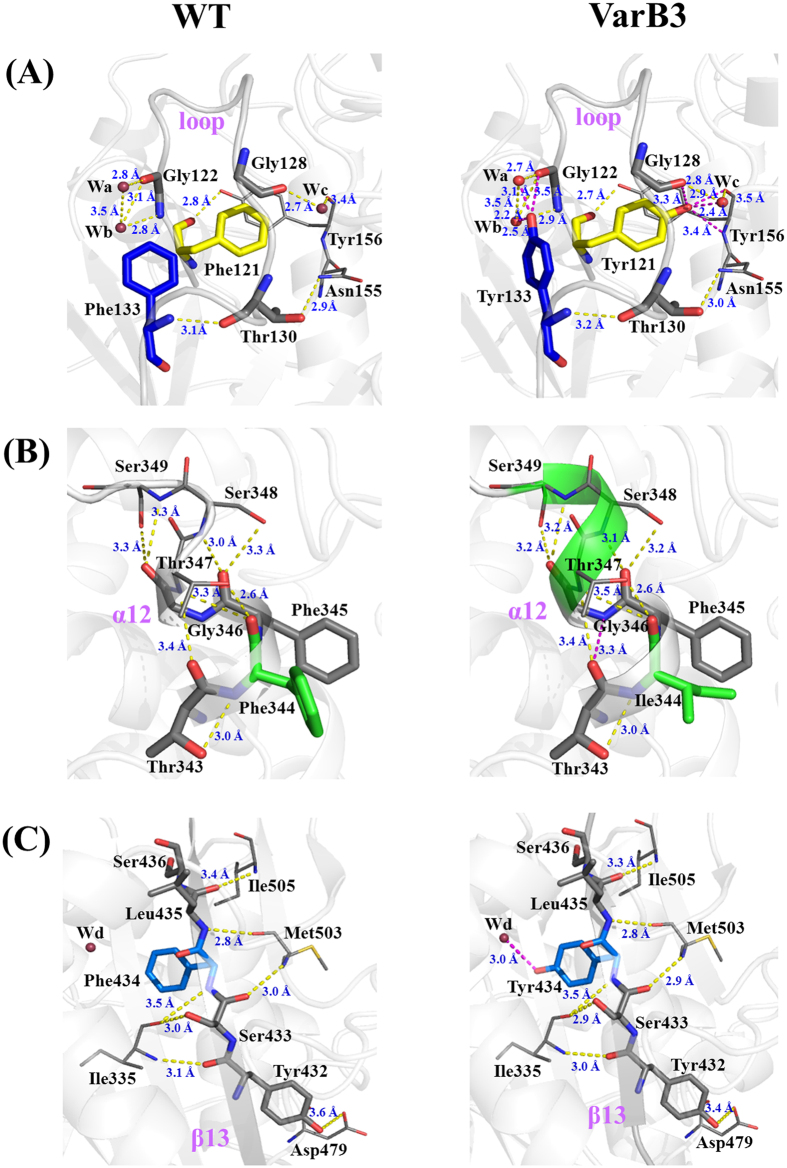
Model structures of WT and the VarB3 mutant (F344I/F434Y/F133Y/F121Y) based on PDB: 1CRL. (**A**) Comparison of the structural changes in the loop region from 120 to 136 which included the mutant position 121 and 133. (**B**) Comparison of α_12_ structural changes around mutant position 344. (**C**) Intramolecular interactions near mutant position 434 in WT and VarB3 mutant. The new generated hydrogen bonds are indicated by purple lines and other hydrogen bonds are represented by yellow dashed lines. Symbols: red, oxygen atom; blue, nitrogen atom.

**Figure 8:**
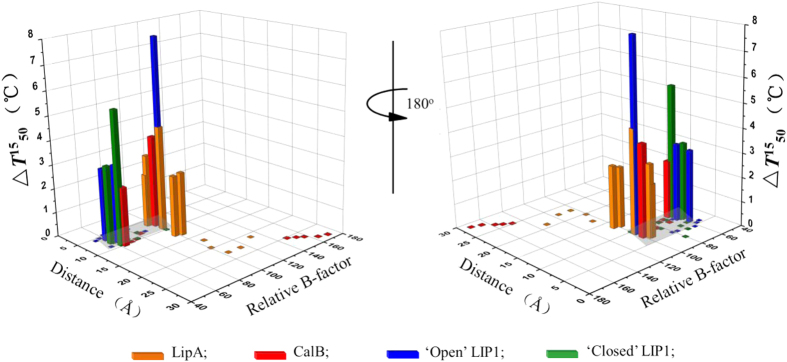
Correlations between the distances of the mutated residues to the catalytic residue serine, relative B-factors, and the thermostabilities 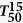 of the lipases. LipA (181 AA); CalB (317 AA); LIP1 (534 AA); the data analysis is based on their mutated results.

The web server PredyFlexy (http://www.dsimb.inserm.fr/dsimb_tools/predyflexy) was used to predict the normalized B-factor based on a library composed of structural prototypes and molecular dynamic simulations. The three dimensional homology models of the targets (Fig. 6B) were constructed using the X-ray structures of the template (PDB code 1CRL). All steps for homology modeling and refinement were accomplished using the program MODELLLER of Discovery Studio 3.5 Client (Accelrys, San Diego, USA). The models with the lowest DOPE score were selected and the accuracy was determined based on the PROCHECK analysis. Hydrogen bonding was assessed using PyMOL, and structural figures were prepared using PyMOL (http://pymol.org).

## Additional Information

**How to cite this article**: Zhang, X.-F. *et al*. A general and efficient strategy for generating the stable enzymes. *Sci. Rep.* **6**, 33797; doi: 10.1038/srep33797 (2016).

## Supplementary Material

Supplementary Information

## Figures and Tables

**Figure 2:**
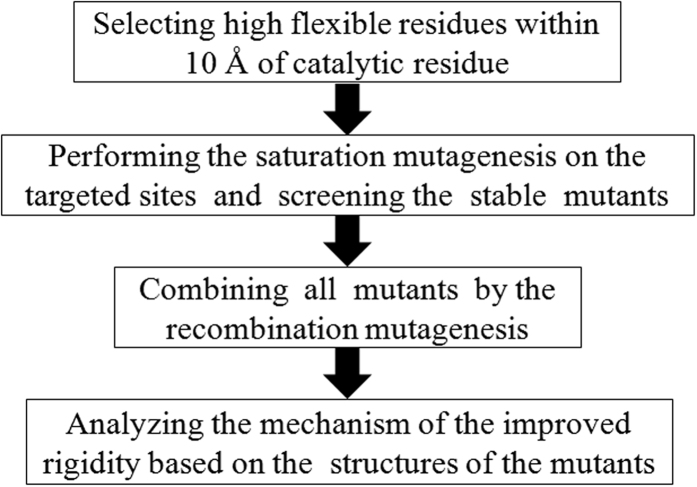
The flow chart of the ACS strategy to improve enzyme stability.

**Table 1:** Enzymatic properties of WT and mutants.

Enzyme	*K*_m_ (μM)	*k*_cat_ (S^−1^)	*k*_cat_/*K*_m_ (μΜ^−1^·S^−1^)	*k*_*d*_ (min^−1^)	*T*_opt_ (^0^C)
WT	13.3 ± 3.2	4,373 ± 273	328	0.12	45
F344M	18.6 ± 3.8	5,691 ± 328	306	0.22 × 10^−1^	55
F344I	14.6 ± 3.0	5,911 ± 315	405	0.29 × 10^−1^	55
F434Y	13.3 ± 2.3	6,075 ± 259	456	0.45 × 10^−1^	50
F133Y	8.5 ± 0.1	3,363 ± 95	396	0.69 × 10^−1^	50
F121Y	16.9 ± 3.6	8,006 ± 457	474	0.89 × 10^−1^	50
VarA1	23.3 ± 5.1	8,924 ± 480	383	0.11 × 10^−1^	57
VarB1	16.1 ± 3.5	11,594 ± 588	724	0.12 × 10^−1^	57
VarA2	10.4 ± 2.7	4,504 ± 277	433	0.46 × 10^−2^	60
VarB2	17.5 ± 4.7	5,671 ± 412	324	0.51 × 10^−2^	60
VarA3	16.9 ± 4.4	3,927 ± 278	232	0.35 × 10^−2^	60
VarB3	16.8 ± 3.9	5,508 ± 352	327	0.29 × 10^−2^	60

## References

[b1] Panke S., Held M. & Wubbolts M. Trends and innovations in industrial biocatalysis for the production of fine chemicals. Curr. Opin. Biotechnol. 15, 272–279 (2004). 15357999 10.1016/j.copbio.2004.06.011

[b2] Andexer J. N., Langermann J. V., Kragl U. & Pohl M. How to overcome limitations in biotechnological processes-examples from hydroxynitrile lyase applications. Trend Biotechnol. 27, 599–608 (2009). 10.1016/j.tibtech.2009.07.005 19716614

[b3] Dicosimo R., McAuliffe J., Poulose A. J. & Bohlmann G. Industrial use of immobilized enzymes. Chem. Soc. Rev. 42, 6347–6474 (2013). 10.1039/c3cs35506c 23436023

[b4] Mamonova T. B., Glyakina A. V., Galzitskaya O. V. & Kurnikova M. G. Stability and rigidity/flexibility—two sides of the same coin? *Biochim*. Biophys. Acta. 1834, 854–866 (2013). 10.1016/j.bbapap.2013.02.011 23416444

[b5] Paredes D. I., Watters K., Pitman D. J., Bystroff C. & Dordick J. S. Comparative void-volume analysis of psychrophilic and mesophilic enzymes: structural bioinformatics of psychrophilic enzymes reveals sources of core flexibility. BMC Struct. Biol. 11, 42 (2011). 22013889 10.1186/1472-6807-11-42 PMC3224250

[b6] Ren W., Steurer M. & Baldwin T. L. Improve the stability and the accuracy of power hardware-in-the-loop simulation by selecting appropriate interface algorithms. IEEE T. Ind. Appl. 44, 1286–1294 (2008).

[b7] Pikkemaat M. G., Linssen A. B. M., Berendsen H. J. C. & Janssen D. B. Molecular dynamics simulations as a tool for improving protein stability. Protein Eng. 15, 185–192 (2002). 11932489 10.1093/protein/15.3.185

[b8] Pan Y., Ma B., Venkataraghavan R. N., Levine A. J. & Nussinov R. In the quest for stable rescuing mutants of p53: computational mutagenesis of flexible loop L1. Biochemistry 44, 1423–1432 (2005). 15683227 10.1021/bi047845y

[b9] Tsou C. Active site flexibility in enzyme catalysis. Enzyme engineering XIV, 1–7 (1998). 10.1111/j.1749-6632.1998.tb10282.x 9928078

[b10] Weng Y., Chang D. T., Huang Y. & Lin C. A study on the flexibility of enzyme active sites. BMC Bioinformatics 12, S32 (2011). 21342563 10.1186/1471-2105-12-S1-S32 PMC3044288

[b11] Parthasarathy S. & Murthy M. Protein thermal stability: insights from atomic displacement parameters (B-values). Protein Eng. 13, 9–13 (2000). 10679524 10.1093/protein/13.1.9

[b12] Yu H. & Huang H. Engieering proteins for thermostability through rigidifying flexible sites. Bitechnol. Adv. 32, 308–315 (2014). 10.1016/j.biotechadv.2013.10.012 24211474

[b13] Reetz M. T., Carballeira J. D. & Vogel A. Iterative saturation mutagenesis on the basis of B-factors as a strategy for increasing protein thermostability. Angew. Chem. Int. Edit. 45, 7745–7751 (2006). 10.1002/anie.200602795 17075931

[b14] Pouderoyen G., Eggert T., Jaeger K. E. & Dijkstra B. W. The crystal structure of *Bacillus subtili* lipase: A minimal α/β hydrolase fold enzyme. J. Microbiol. Biotechnol. 309, 215–226 (2001). 10.1006/jmbi.2001.4659 11491291

[b15] Zhang J. . High-throughput screening of B-factor saturation mutated *Rhizomucor miehei* lipase thermostability based on synthetic reaction. Enzyme Microb. Tech. 50, 325–330 (2012). 10.1016/j.enzmictec.2012.03.002 22500900

[b16] Damnjanović J., Takahashi R., Suzuki A., Nakano H. & Iwasaki Y. Improving thermostability of phosphatidylinositol-synthesizing *streptomyces* phospholipase D. Protein Eng. Des. Sel. 25, 415–424 (2012). 22718790 10.1093/protein/gzs038

[b17] Rose G. D., Geselowitz A. R., Lesser G. J., Lee R. H. & Zehfus M. H. Hydrophobicity of amino acid residues in globular proteins. Science 229, 834–838 (1985). 4023714 10.1126/science.4023714

[b18] Tsou C. Conformational flexibility of enzyme active sites. Science 262, 380–381 (1993). 8211158 10.1126/science.8211158

[b19] Uppenberg J., Hansen M. T., Patkar S. & Jones T. A. The sequence, crystal structure determination and refinement of two crystal forms of lipase B from *Candida antarctica*. Structure 2, 293–308 (1994). 8087556 10.1016/s0969-2126(00)00031-9

[b20] Xie Y. . Enhanced enzyme kinetic stability by increasing rigidity within the active site. J. Biol. Chem. 289, 7994–8006 (2014). 24448805 10.1074/jbc.M113.536045 PMC3953309

[b21] Grochulski P. . Insights into interfacial activation from an open structure of *Candida rugosa* lipase. J. Biol. Chem. 268, 12843–12847 (1993). 8509417

[b22] Grochulski P., Li Y., Schrag J. D. & Cygler M. Two conformational states of *Candida rugosa* lipase. Protein Sci. 3, 82–91 (1994). 8142901 10.1002/pro.5560030111 PMC2142478

[b23] Chang S. W., Lee G. C. & Shaw J. F. Codon optimization of *Candida rugosa* LIP1 gene for improving expression in *Pichia pastoris* and biochemical characterization of the purified recombinant LIP1 lipase. J. Agr. Food chem. 54, 815–822 (2006). 16448188 10.1021/jf052183k

[b24] Zhang X. . Modulation of the thermostability and substrate specificity of *Candida rugosa* lipase1 by altering the acyl-binding residue Gly414 at the α-helix-connecting bend. Enzyme Microb. Tech. 82, 34–41 (2016). 10.1016/j.enzmictec.2015.08.006 26672446

[b25] Reetz M. T. & Carballeira J. D. Iterative saturation mutagenesis (ISM) for rapid directed evolution of functional enzymes. Nat. Protoc. 2, 891–903 (2007). 17446890 10.1038/nprot.2007.72

[b26] Kouker G. & Jaeger K. E. Specific and sensitive plate assay for bacterial lipases. Appl. Environ. Microbiol. 53, 211–213 (1987). 3103532 10.1128/aem.53.1.211-213.1987 PMC203632

[b27] Ahmad S., Kamal M. Z., Sankaranarayanan R. & Rao N. M. Thermostable *Bacillus subtilis* lipase: *in vitro* evolution and structural insight. J. Mol. Biol. 381, 324–340 (2008). 18599073 10.1016/j.jmb.2008.05.063

[b28] Bosshart A., Panke S. & Bechtold M. Systematic optimization of interface interactions increases the thermostability of a multimeric enzyem. Angew.Chem. Int. Ed. 52, 9673–9676 (2013). 10.1002/anie.201304141 23893529

[b29] Kille S. . Reducing codon redundancy and screening effort of combinatorial protein libraries created by saturation mutagenesis. ACS Synth. Biol. 2, 83–92 (2012). 23656371 10.1021/sb300037w

[b30] Bosley A. D. & Ostermeier M. Mathematical expressions useful in the construction, description and evaluation of protein libraries. Biomol. Eng. 22, 57–61 (2005). 15857784 10.1016/j.bioeng.2004.11.002

[b31] Patrick W. M., Firth A. E. & Blackburn J. M. User-friendly algorithms for estimating completeness and diversity in randomized protein-encoding libraries. Protein Eng. 16, 451–457 (2003). 12874379 10.1093/protein/gzg057

[b32] Brevern A. G., Bornot A., Craveur P., Etchebest C. & Gelly J. C. Predyflexy: Flexibility and local structure prediction from sequence. Nucleic Acids Res. 40, W317–W322 (2012). 22689641 10.1093/nar/gks482 PMC3394303

[b33] Laskowski R. A., Macarthur M. W., Moss D. S. & Thornton J. M. Procheck: a program to check the stereochemical quality of protein structures. J. Appl. Cryst. 26, 283–291 (1993).

[b34] Lee J., Suh S. W. & Shin S. Computational studies of essential dynamics of *Pseudomonas cepacia* lipase. J. Biomol. Struct. Dyn. 18, 297–309 (2000). 11089650 10.1080/07391102.2000.10506667

[b35] Toyoshima C. & Nomura H. Structural changes in the calcium pump accompanying the dissociation of calcium. Nature 418, 605–611 (2002). 12167852 10.1038/nature00944

[b36] Pace C. N. . Tyrosine hydrogen bonds make a large contribution to protein stability. J. Mol. Biol. 312, 393–404 (2001). 11554795 10.1006/jmbi.2001.4956

[b37] Myers J. K. & Pace C. N. Hydrogen stabilizes globular protein. Biophys. J. 71, 2033–2039 (1996). 8889177 10.1016/S0006-3495(96)79401-8 PMC1233669

[b38] Yang H. . Structure-based engineering of methionine residues in the catalytic cores of alkaline amylase from *Alkalimonas amylolytica* for improved oxidative stability. Appl. Environ. Microbiol. 78, 7519–7526 (2012). 22865059 10.1128/AEM.01307-12 PMC3485717

[b39] Duan X., Chen J. & Wu J. Improving the thermostability and catalytic efficiency of *Bacillus deramificans* pullulanase by site-directed mutagenesis. Appl. Environ. Microb. 79, 4072–4077 (2013). 10.1128/AEM.00457-13 PMC3697558 23624477

[b40] Wang X., Minasov G. & Shoichet B. K. Evolution of an antibiotic resistance enzyme constrained by stability and activity trade-offs. J. Mol. Biol. 320, 85–95 (2002). 12079336 10.1016/S0022-2836(02)00400-X

[b41] Dick M. . Trading off stability against activity in extermophilic aldolases. Sci. Rep. 6, 17908 (2016). 26783049 10.1038/srep17908 PMC4725968

[b42] Duan X., Chen J. & Wu J. Improcing the thermostability and catalytic efficiency of *Bacillus deramificans* pullulanase by site-directed mutagenesis. Appl. Environ. Microbiol. 79, 4072–4077 (2013). 23624477 10.1128/AEM.00457-13 PMC3697558

[b43] Lu Z., Wang Q., Jiang S., Zhang G. & Ma Y. Truncation of the unique N-terminal domain improved the thermos-stability and specific activitity of alkaline α-amylase Amy703. Sci. Rep. 6, 22465 (2016). 26926401 10.1038/srep22465 PMC4772547

[b44] Kim D. & Guengerich F. P. Random mutagenesis by whole-plasmid PCR amplification. Methods Mol. Biol. 192, 241–245 (2002). 12494656 10.1385/1-59259-177-9:241

[b45] Niu W., Li Z., Zhang D., Yu M. & Tan T. Improved thermostability and the optimum temperature of *Rhizopus arrhizus* lipase by directed evolution. J. Mol. Catal. B-Enzym. 43, 33–39 (2006).

[b46] Smith P. K. . Measurement of protein using bicinchoninic acid. Anal. Biochem. 150, 76–85 (1985). 3843705 10.1016/0003-2697(85)90442-7

[b47] Wahler D. & Reymond J. L. Novel methods for biocatalyst screening. Curr. Opin. Chem. Biol. 5, 152–158 (2001). 11282341 10.1016/s1367-5931(00)00184-8

[b48] Winkler U. K. & Stuckmann M. Glycogen, hyaluronate, and some other polysaccharides greatly enhance the formation of exolipase by *Serratia marcescens*. J. Bacteriol. 138, 663–670 (1979). 222724 10.1128/jb.138.3.663-670.1979 PMC218088

[b49] Singh A. K. & Chhatpar H. S. Purification, characterization and thermodynamics of antifungal protease from *Streptomyces* sp. A6. J. Basic. Microb. 51, 424–432 (2011). 10.1002/jobm.201000310 21656799

