# Supplementary material for: A general and efficient strategy for generating the stable enzymes
Source: Sci Rep. 2016 Sep 26;6:33797. doi: 10.1038/srep33797 (PMC5036031; doi:10.1038/srep33797)
Supplement: Supplementary Information [file srep33797-s1.pdf]

Supplementary material

A general and efficient strategy for generating the stable enzymes

Xiao-Fei Zhang[1], Guang-Yu Yang[1], Yong Zhang[1], Yuan Xie[1], Stephen G. Withers[2], Yan Feng[1*]

[1] State Key Laboratory of Microbial Metabolism, School of Life Sciences and Biotechnology, Shanghai Jiao Tong University, Shanghai 200240, China.

[2] Department of Chemistry, University of British Columbia Vancouver, British Columbia V6T 1Z1, Canada.

[*]Correspondence and requests for materials should be addressed to Y.F. (email: yfeng2009@sjtu.edu.cn)

Table S1. Oligonucleotide primers used for saturation mutagenesis of LIP1.

Library	NNK	Sequence（5’→3’）
A-Forward	344, 345	GAGGGCACC**NNKNNK**GGCACCTCTTCT
A-Reverse		GGTGCC**MNNMNN**GGTGCCCTCGTCGTTCT
B-Forward	302	TACTCCTCG**NNK**CGGTTGTCTTACCTCCC
B-Reverse		GACAACCG**MNN**CGAGGAGTACGCCAAGA
C-Forward	133	CACCAGCACC**NNK**CCTCCCGCCCAGATGA
C-Reverse		GGGCGGGAGG**MNN**GGTGCTGGTGCCACC
D-Forward	124,125	CGGC**NNKNNK**GAGGTGGGTGGCACCA
D-Reverse		CACCCACCTC**MNNMNN**GCCGCCAAAGA
E-Forward	126, 127	CGGGTTT**NNKNNK**GGTGGCACCAGCA
E-Reverse		GCCACC**MNNMNN**AAACCCGCCGCCAAA
F-Forward	121	TCTGGATC**NNK**GGCGGCGGGTTTGAGG
F-Reverse		CCGCCGCC**MNN**GATCCAGAGCATCACC
G-Forward	87, 88	CAGTCCAAGGTG**NNKNNK**GCGGTGTCTCCG
G-Reverse		AGACACCGC**MNNMNN**CACCTTGGACTGCAT
H-Forward	122, 123	CTGGATCTTT**NNKNNK**GGGTTTGAGGTGGGT
H-Reverse		CCACCTCAAACCC**MNNMNN**AAAGATCCAGAG
I-Forward	84, 85	CTTGGTGATGCAG**NNKNNK**GTGTTTGAGGCG
I-Reverse		CCTCAAACAC**MNNMNN**CTGCATCACCAAGTC
J-Forward	296	CACCCCTGGG**NNK**TTGGCGTACTCCTCGTTG
J-Reverse		GGAGTACGCCAA**MNN**CCCAGGGGTGTTGTT
K-Forward	434	CAAGTACTCA**NNK**CTCTCTAAGCAGCTCTCT
K-Reverse		GCTGCTTAGAGAG**MNN**TGAGTACTTGGTGCC
L-Forward	414	GCGACCTT**NNK**TTTACGCTTGCTC
L-Reverse		AAGCGTAAA**MNN**AAGGTCGCCGAG

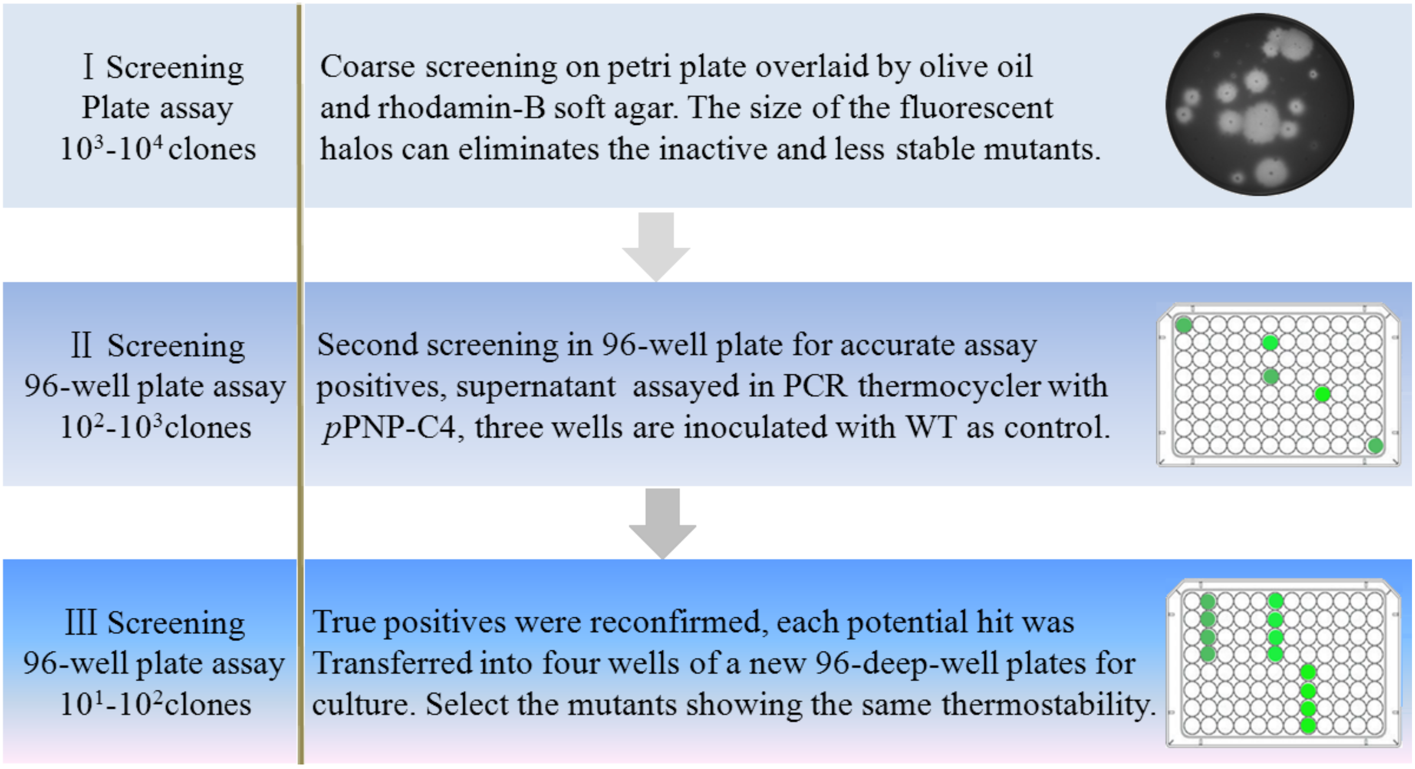

Figure S1. Three-tier procedure for selecting the mutants with high thermostability and catalytic activity.

Table S2. Thermostabilizing mutations isolated by three-tier screening and ordered recombination mutagenesis.

Mutants	Codon change	Amnio acid change
F121Y	TTT121TAT	Phe121Tyr
F344I	TTC344ATT	Phe344Ile
F344M	TTC344ATG	Phe344Met
F133Y	TTC133TAT	Phe133Tyr
F434Y	TTC434TAT	Phe434Tyr
VarA1	TTC344ATT/TTC434TAT	Phe344Ile/Phe434Tyr
VarB1	TTC344ATG/TTC434TAT	Phe344Met/Phe434Tyr
VarA2	TTC344ATT/TTC434TAT/TTC133TAT	Phe344Ile/Phe434Tyr/Phe133Tyr
VarB2	TTC344ATG/TTC434TAT/TTC133TAT	Phe344Met/Phe434Tyr/Phe133Tyr
VarA3	TTC344ATT/TTC434TAT/TTC133TAT /TTT121TAT	Phe344Ile/Phe434Tyr/Phe133Tyr /Phe121Tyr
VarB3	TTC344ATG/TTC434TAT/TTC133TAT /TTT121TAT	Phe344Met/Phe434Tyr/Phe133Tyr /Phe121Tyr

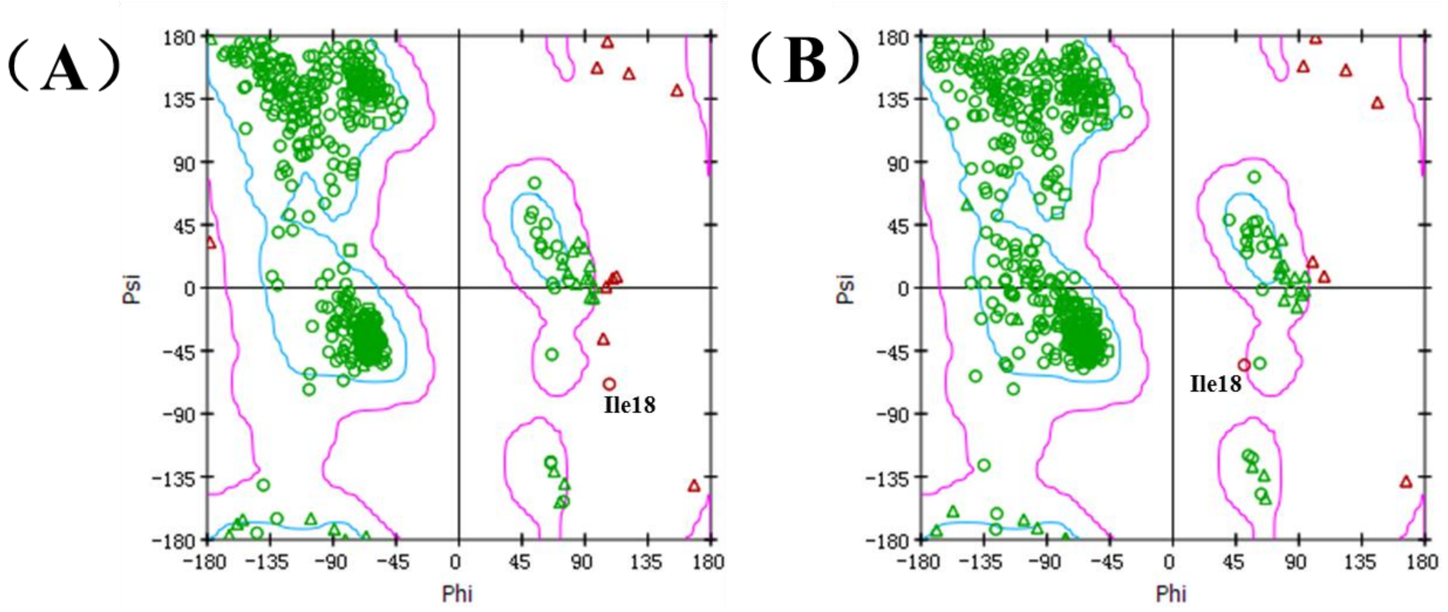

Figure S2. The Ramachandran Plots of the VarB3 mutant (A) and WT (B). Red triangle represents the glycine in the disallowed regions. Red circle represents reside Ile18 in the disallowed regions.

Table S3. Prediction of normalized B-factor of flexible residues within active center from all mutants.

Residues	Normalized B-factor											
	**WT**	**F121Y**	**F133Y**	**F434Y**	**F344I**	**F344M**	**VarB1**	**VarB2**	**VarB3**	**VarA1**	**VarA2**	**VarA3**
84	0.377	0.827	**0.207**	0.827	0.393	0.393	0.393	0.827	0.393	**0.219**	0.601	**0.207**
85	0.546	**0.148**	**0.424**	**0.026**	**0.148**	**0.148**	**0.026**	**0.148**	**0.148**	**0.424**	0.546	**0.026**
87	0.509	**0.344**	0.509	**0.344**	0.509	**0.344**	0.509	**0.344**	0.509	0.509	0.509	0.509
88	0.648	0.648	0.657	0.665	0.657	0.665	0.665	0.665	0.648	0.648	0.648	0.657
121	0.142	0.142	0.142	0.142	0.142	0.142	0.142	0.142	0.142	0.142	0.142	0.142
122	0.774	0.774	0.774	0.774	0.774	0.774	0.774	0.774	0.933	0.774	0.774	0.933
123	0.78	0.877	0.907	0.907	0.907	0.907	0.877	0.907	0.818	0.907	0.907	0.818
124	0.753	**0.476**	**0.613**	**0.613**	0.613	**0.613**	**0.585**	**0.613**	**0.585**	**0.613**	**0.585**	**0.585**
125	0.083	0.158	0.083	0.083	0.083	0.083	0.083	0.083	0.097	0.083	0.083	0.097
126	0.136	**-0.137**	**0.065**	0.136	**-0.137**	**-0.137**	0.136	**0.065**	**-0.137**	**-0.137**	**0.065**	**-0.137**
127	0.296	0.296	0.296	0.296	0.296	0.296	0.358	0.296	0.387	0.296	0.296	0.387
133	0.689	0.689	**0.618**	**0.461**	**0.618**	**0.618**	**0.66**	**0.525**	0.689	**0.618**	**0.618**	0.689
296	0.409	0.409	0.409	0.409	0.548	0.548	0.548	0.548	0.409	0.548	0.409	0.409
302	-0.223	-0.223	-0.223	-0.223	-0.223	-0.223	-0.223	-0.223	-0.223	-0.223	-0.223	-0.002
344	-0.147	-0.147	-0.147	-0.147	**-0.082**	-0.147	-0.147	-0.147	-0.147	-0.147	-0.147	-0.147
345	-0.401	-0.401	-0.401	-0.401	-0.351	-0.401	**-0.468**	**-0.468**	**-0.468**	**-0.468**	**-0.468**	**-0.468**
414	0.002	0.124	0.126	0.002	0.144	0.144	0.002	0.124	0.002	0.002	0.002	0.002
434	-0.403	-0.403	-0.403	**-0.581**	-0.322	-0.262	**-0.581**	-0.379	**-0.581**	**-0.581**	**-0.581**	**-0.581**

Red represents the lower normalized B-factors of variants than those of WT.

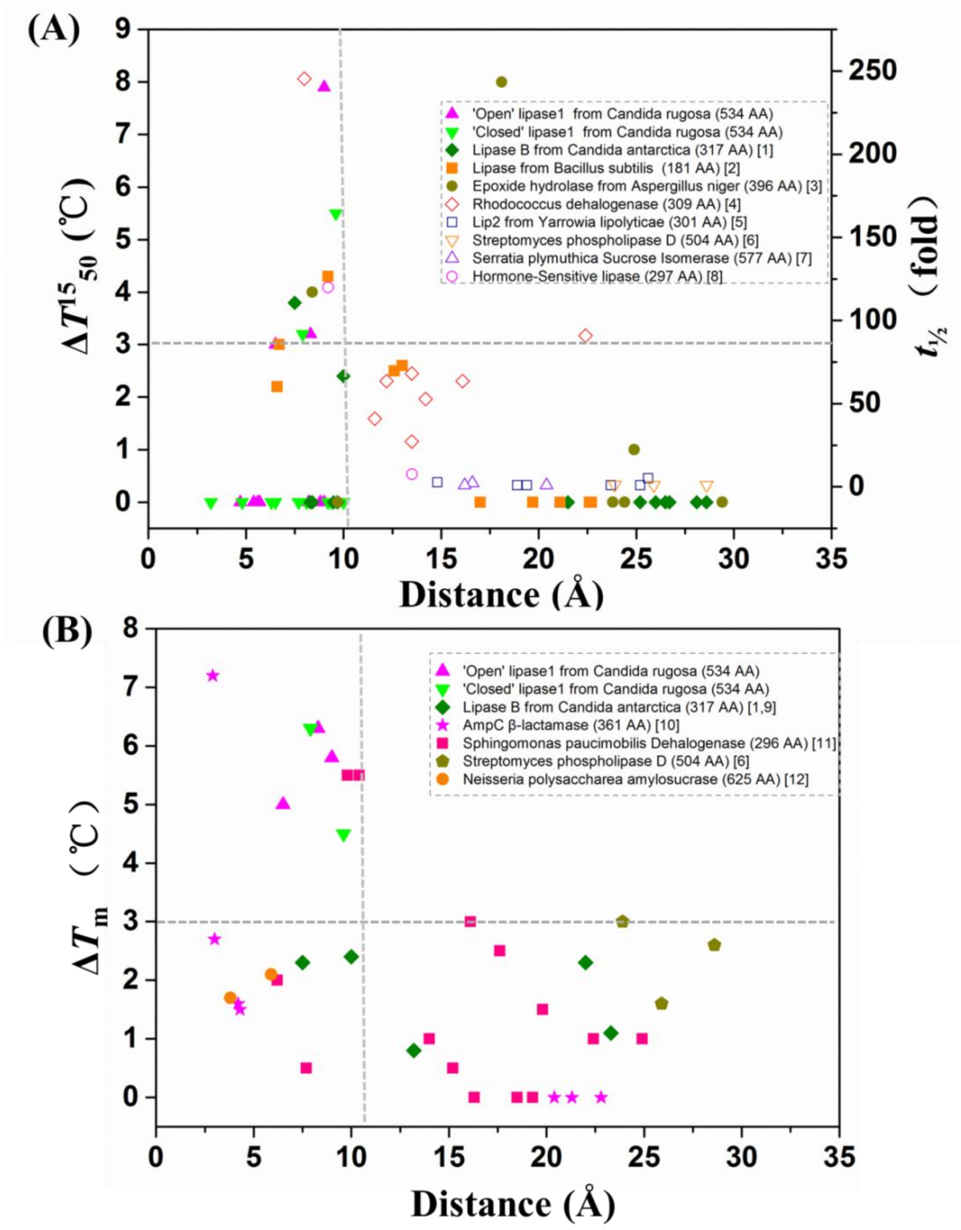

Fig. S3. Correlations between the distance of the mutated residues to the catalytic residues and the stability. (A) correlations between the distance and the thermostabilities ΔT^{15}_{50} (solid) and $t_{1/2}$ (hollow). (B) Correlations between the distance and the thermodynamic ΔT_m. The gray dashes lines represent boundary and separated the mutagenesis sites into the different regions. The PDB codes of the enzymes analyzed are *C. rugosa lipase1* (1CRL, 1TRH), *C. antarctica* lipase B (1TCA), *Bacillus subtilis* lipase (1ISP), *Aspergillus niger* epoxide hydrolase (1QO7), *Rhodococcus rhodochrous* dehalogenase (1CQW), *Yarrowia lipolytica* Lip2 (3O0D), *Streptomyces* phospholipase D (2ZE4), *Serratia plymuthica* Sucrose Isomerase (3GBD), Hormone-Sensitive Lipase (4XVC), AmpC b-lactamase

(1KE4), *Sphingomonas paucimobilis* dehalogenase (1MJ5), *Neisseria polysaccharea* amylosucrase (1G5A).

Reference:

[1] Xie, Y. *et al*. Enhanced enzyme kinetic stability by increasing rigidity within the active site. *J. Biol. Chem*. **289**, 7994-8006 (2014).

[2] Reetz, M. T., Carballeira, J. D. & Vogel, A. Iterative saturation mutagenesis on the basis of B-factors as a strategy for increasing protein thermostability. *Angew. Chem. Int. Edit*. **45**, 7745-7751 (2006).

[3] Gumulya, Y. & Reetz, M. T. Enhancing the thermal robustness of an enzyme by directed evolution: least favorable starting points and inferior mutants can map superior evolutionary pathways. *ChemBioChem*.**12**, 2502-2510 (2011).

[4] Gray, K. A. *et al*. Rapid evolution of reversible denaturation and elevated melting temperature in a microbial haloalkane dehalogenase. *Adv. Synth. Catal*. **343**, 607-617 (2001).

[5] Wen, S., Tan, T. & Zhao H. Improving the thermostability of lipase Lip2 from *Yarrowia lipolytica*. *J. biotech*. **164**, 248-253 (2013).

[6] Damnjanović, J., Takahashi, R., Suzuki, A., Nakano, H. & Iwasaki, Y. Improving thermostability of phosphatidylinositol-synthesizing *Streptomyces* phospholipase D. *Protein Eng. Des. Sel*. **25**, 415-424 (2012).

[7] Duan, X., Cheng, S., Ai, Y. & Wu, J. Enhancing the Thermostability of *Serratia plymuthica* Sucrose Isomerase Using B-Factor-Directed Mutagenesis. *PloS one*, **11**, e0149208 (2016).

[8] Li, P. *et al*. Interdomain Hydrophobic Interactions Modulate the Thermostability of Microbial Esterases from the Hormone-Sensitive Lipase Family. *J. Mol. Biol*. **290**, 11188-11198 (2015).

[9] Kim, H. S., Le, Q. A. T. & Kim, Y. H. Development of thermostable lipase B from *Candida antarctica* (CalB) through in silico design employing B-factor and RosettaDesign. *Enzyme Microb. Tech*. **47**, 1-5 (2010).

[10] Beadle, B. M. & Shoichet, B. K. Structural bases of stability–function tradeoffs in enzymes. *J. Mol. Biol*. **321**, 285-296 (2002).

[11] Floor, R. J. *et al*. Computational library design for increasing *haloalkane dehalogenase* stability. *ChemBioChem*, **15**, 1660-1672, (2014).

[12] Daudé, D., Topham, C. M., Remaud-Siméon, M. & André. Probing impact of active site residue mutations on stability and activity of Neisseria *polysaccharea amylosucrase*. *Protein Sci*. **22**, 1754-1765 (2013).
